# CIRCprimerXL: Convenient and High-Throughput PCR Primer Design for Circular RNA Quantification

**DOI:** 10.3389/fbinf.2022.834655

**Published:** 2022-03-01

**Authors:** Marieke Vromman, Jasper Anckaert, Jo Vandesompele, Pieter-Jan Volders

**Affiliations:** ^1^ OncoRNALab, Cancer Research Institute Ghent (CRIG), Ghent, Belgium; ^2^ Department of Biomolecular Medicine, Ghent University, Ghent, Belgium

**Keywords:** RT-qPCR – real-time quantitative polymerase chain reaction, circular RNA (circRNA), primer design tool, back-splice junction, circRNA validation

## Abstract

Circular RNA (circRNA) is a class of endogenous non-coding RNA characterized by a back-splice junction (BSJ). In general, large-scale circRNA BSJ detection is performed based on RNA sequencing data, followed by the selection and validation of circRNAs of interest using RT-qPCR with circRNA-specific PCR primers. Such a primer pair is convergent and functional on the circRNA template but divergent and non-functional on the linear host gene. Although a few circRNA primer design pipelines have been published, none of them offer large-scale, easy-to-use circRNA primer design. Other limitations are that these tools generally do not take into account assay specificity, secondary structures, and SNPs in the primer annealing regions. Furthermore, these tools are limited to circRNA primer design for humans (no other organisms possible), and no wet-lab validation is demonstrated. Here, we present CIRCprimerXL, a circRNA RT-qPCR assay design pipeline based on the primer design framework primerXL. CIRCprimerXL takes a circRNA BSJ position as input, and designs BSJ-spanning primers using Primer3. The user can choose to use the unspliced or spliced circRNA sequence as template. Prior to primer design, sequence regions with secondary structures and common SNPs are flagged. Next, the primers are filtered based on predicted specificity and the absence of secondary structures of the amplicon to select a suitable primer pair. Our tool is both available as a user-friendly web tool and as a stand-alone pipeline based on Docker and Nextflow, allowing users to run the pipeline on a wide range of computer infrastructures. The CIRCprimerXL Nextflow pipeline can be used to design circRNA primers for any species by providing the appropriate reference genome. The CIRCprimerXL web tool supports circRNA primer design for human, mouse, rat, zebrafish, *Xenopus tropicalis,* and *C. elegans*. The design process can easily be scaled up for the qPCR assay design of tens of thousands of circRNAs within a couple of hours. We show how CIRCprimerXL has been successfully used to design qPCR assays for over 15,000 human circRNAs of which 20 were empirically validated. CIRCprimerXL software, documentation, and test data can be found at: https://github.com/OncoRNALab/CIRCprimerXL. CIRCprimerXL is also implemented as a webtool at: https://circprimerxl.cmgg.be.

## 1 Introduction

**FIGURE 1:**
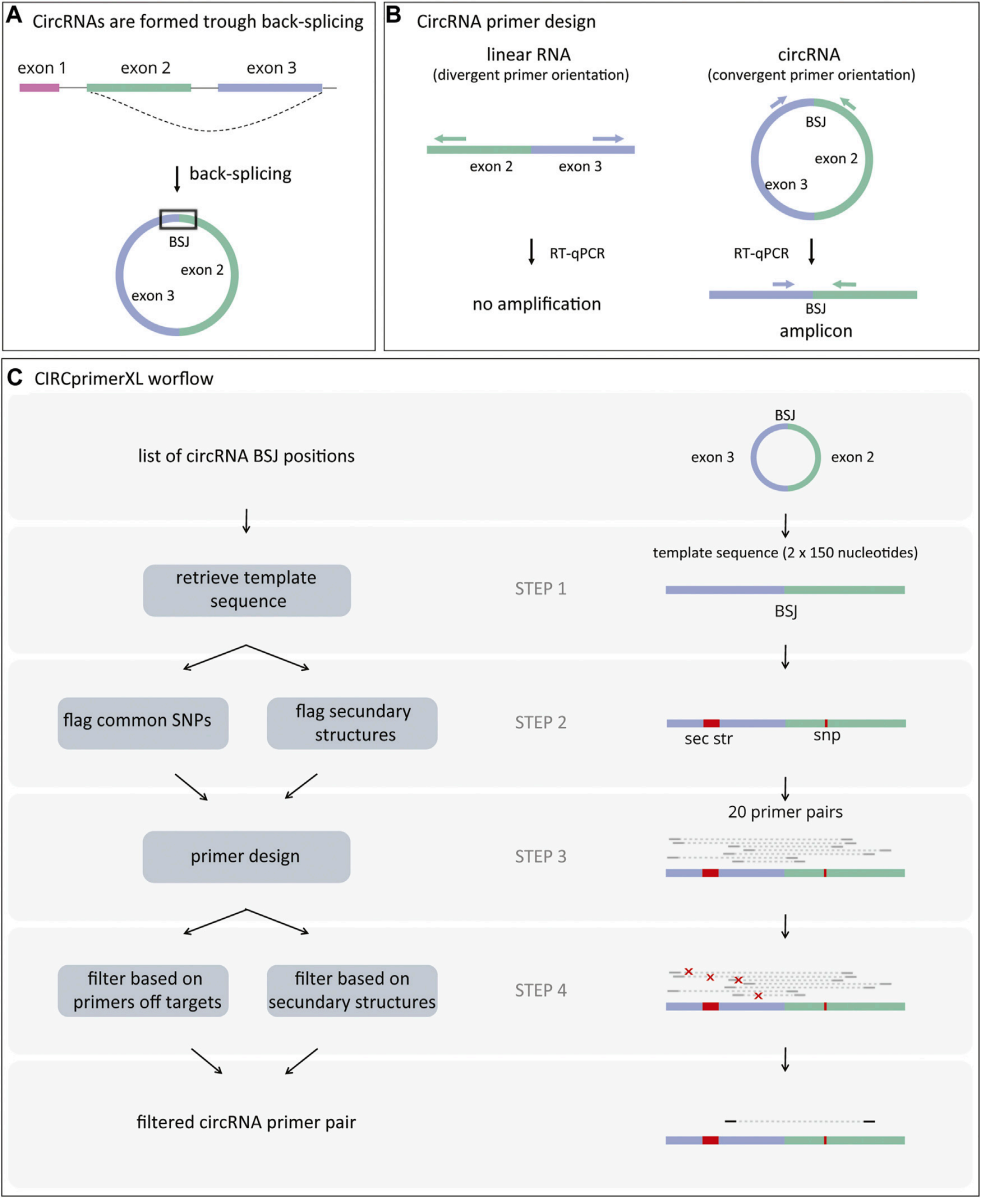
CircRNAs formation and primer design. **(A)** CircRNAs are formed through a process called back-splicing, which results in a covalently closed loop characterized by a back-splice junction (BSJ). **(B)** CircRNA-specific primers amplify the region surrounding the BSJ when annealing to the circRNA (convergent orientation). These primers will not amplify linear RNA (divergent orientation). **(C)** First, CIRCprimerXL retrieves the sequence surrounding the circRNA BSJ (step 1). Next, both common SNPs and regions with secondary structures are flagged (step 2) and avoided during primers design (step 3). Finally, the primers are filtered based on their specificity and secondary structures in the amplicon (step 4).

Circular RNA (circRNA) is a class of endogenous non-coding RNA that is widespread and abundant in many organisms. Although the exact function and working mechanism are lacking for the majority, some circRNAs are aberrantly expressed in pathological conditions such as cancer (Meng et al., 2017; Lux and Bullinger 2018). CircRNAs are formed through back-splicing, a non-canonical form of alternative splicing. Back-splicing results in a covalently closed loop characterized by a non-linear back-splice junction (BSJ) between a splice donor and an upstream splice acceptor (Figure 1A) (Salzman et al., 2012). The detection and quantification of circRNAs is based on the presence of their characteristic BSJ.

In general, large-scale circRNA detection is performed based on RNA sequencing data, using specific circRNA detection algorithms (Gao and Zhao 2018; Hansen et al., 2015; Zeng et al., 2017; Jakobi and Dieterich 2019). The circRNAs of interest are then confirmed by reverse transcription quantitative polymerase chain reaction (RT-qPCR) after ribonuclease R (RNase R) treatment (Vromman et al., 2021). RNase R is an enzyme that degrades linear RNA and thus reveals false-positive circRNA signals originating from endogenous *trans*-splicing (Vincent and Deutscher 2006; Pfafenrot and Preußer 2019). For RT-qPCR validation, circRNA-specific PCR primers are required (Figure 1B). Since these primers have a divergent orientation on the linear template, the linear host-gene will not be detected. Although a few circRNA primer design pipelines have been published, none of them offer large-scale, easy-to-use circRNA primer design (Dudekula et al., 2016; Jakobi, Uvarovskii, and Dieterich 2018; Panda and Gorospe 2018; Zhong et al., 2018). Other limitations are that these tools generally do not take into account assay specificity, spliced/unspliced nature of template, secondary structures, and SNPs in the primer annealing regions, and that no wet-lab validation is demonstrated. Here, we present CIRCprimerXL, a high-throughput and user-friendly circRNA qPCR assay design pipeline and web interface based on the primer design framework primerXL (Lefever et al., 2017).

## 2 Methods

### 2.1 Implementation

The CIRCprimerXL scripts are written in Python (version 3.8.10). The pipeline is written using Nextflow (version 20.10.0.5430) and runs in a Docker container (version 20.10.11).

CIRCprimerXL is also implemented as a web application. The backend of the web application is built with Python (version 3.8-slim-buster) and the Flask framework (version 2.0.2), while the frontend is based on Bootstrap (version 5.1.). CIRCprimerXL is therefore compatible with the following web browsers: Chrome, Opera, Microsoft Edge, Firefox, and Safari.

### 2.2 CIRCprimerXL Workflow

A general overview of the pipeline can be found in Figure 1C.

STEP 1: The sequence around the BSJ is retrieved from a reference genome (default: homo sapiens) using fastahack (https://github.com/ekg/fastahack, RRID:SCR_016090, version 1.0). If both the start and end position of the circRNA are known splice sites or overlap with a known exon, the introns are removed from the template sequence based on an exon annotation file (default: Ensembl GTF, version GRCh38.104). If a list of canonical transcripts and/or transcripts of interest is provided (format: ENST transcript ids), it is used for transcript selection (default: RefSeq canonical transcripts, version GRCh38. v0.95). Alternatively, if the start and/or end position of the circRNA are intronic, exon-intron boundaries and intronic regions are included in the template sequence. By default, 150 nucleotides on both sides of the BSJ are concatenated to form the 300-nucleotide template sequence. If a circRNA is smaller than the requested template size, the template size is reduced to the circRNA size.
STEP 2: All common SNPs present in the template sequence are retrieved and flagged for exclusion (default SNP database: http://hgdownload.soe.ucsc.edu/gbdb/hg38/snp/dbSnp153Common.bb). The template sequence is screened for secondary structures using NUPACK (version 4.0.0.23) (Fornace, Porubsky, and Pierce 2020). All nucleotides involved in a secondary structure are flagged.
STEP 3: Twenty (by default) primer pairs are designed for each circRNA using Primer3 RRID:SCR_003139, version 2.5.0) (Untergasser et al., 2012). For this design, the Primer3 settings previously optimized in primerXL are used (Lefever et al., 2017). The most important parameters are primer length (nucleotides) = 16-20-30 (min-opt-max); primer Tm: 58-59-60 (min-opt-max); max Tm difference: 2°C; primer GC %: 30-50-80 (min-opt-max); concentration of monovalent cations: 50 mM; concentration of divalent cations: 3 mM; amplicon product size: 50-250 nucleotides. Furthermore, the nucleotides flagged for SNPs and secondary structures from STEP 2 are avoided.
STEP 4: Each primer pair is then screened for off-target activity by mapping the primer pair to a reference transcriptome (default: human cDNA and ncRNA) using bowtie (RRID:SCR_005476, version 1.3.0) (Langmead et al., 2009). When a match is found in the reference transcriptome, the primer pair is discarded, unless there are multiple mismatches present in both primers (Table 1) (Lefever et al., 2013). Simultaneously, the amplicon is screened for possible secondary structure formation using NUPACK (Fornace, Porubsky, and Pierce 2020), as this can prevent efficient amplification (Hoebeeck et al., 2005). If the amplicon has a secondary structure with a deltaG (Gibbs free energy) lower than −15 kcal/mol, the primer pair is rejected. If there is a secondary structure present with a deltaG between −15 kcal/mol and −5 kcal/mol, but the secondary structure is not in the primer sequence, or the delta G is higher than −5 kcal/mol, the primer pair is not rejected. The list of filtered primers is then sorted by increasing amplicon size. The selected primer pair is the one with the smallest amplicon size, as it has a higher chance of amplifying the BSJ sequence (smaller chance of priming to introns or neighboring exons).

**TABLE 1:** Tolerated off-targets for circRNA primers (based on (Lefever et al., 2013)). When the specificity filter of CIRCprimerXL is set to “strict” (default), primers with predicted off-targets are discarded, unless there are at least 4 mismatches for a single primer or a total of at least 5 mismatches between the primers and the potential off-target sequence. Alternatively, the specificity setting can be set to “loose”, where at least 3 mismatches for a single primer or a total of at least 4 mismatches between the primers and the potential off-targets are also allowed.

Number of mismatches in primer 1	Number of mismatches in primer 2	Sum of the number of mismatches	Specificity filter setting	
			Strict	Loose
0	0	0	off-target	off-target
0	1	1	off-target	off-target
1	1	2	off-target	off-target
2	0	2	off-target	off-target
2	1	3	off-target	off-target
3	0	3	off-target	no off-target
3	1	4	off-target	no off-target
2	2	4	off-target	no off-target
4	0	4	no off-target	no off-target
2	3	5	no off-target	no off-target
3	3	6	no off-target	no off-target

Finally, a list containing a final primer pair for each circRNA is compiled and returned to the user, along with the complete list of evaluated primers (more than one primer pair can pass all filters) and a report showing primer design rates and reasons for failure.

### 2.3 Performance Tests

To evaluate the performance of CIRCprimerXL, a circRNA dataset was generated by detecting circRNAs with two different circRNA detection tools, find_circ (Memczak et al., 2013) and CIRCexplorer2 (RRID:SCR_021664) (Zhang et al., 2016) in a deeply sequenced total RNA from SW480 colon carcinoma cells (SRA: SRS11316475). From this circRNA dataset, a random subset of 2000 circRNAs was used to investigate the primer design success rate of CIRCprimerXL. To evaluate the scalability and run time of CIRCprimerXL, the pipeline was used to design primers for a random subset of 15,000 circRNAs.

### 2.4 Computer System’s Specifications

For smaller runs (up to 200 circRNAs), CIRCprimerXL was run on a standard 64-bit laptop computer running macOS (Monterey 12.0.1), equipped with 16 GB of memory, and 2.5 GHz Dual-Core Intel Core i7 processor. For larger runs (up to 15,000 circRNAs), a high-performance cluster (HPC) (Linux) was used (requesting 40 GB memory and 16 CPUs).

### 2.5 RT-qPCR Validation

#### 2.5.1 Primer Efficiency

Primer efficiency was tested using a 6-point 10-fold dilution series of synthetic DNA template positive controls, previously described in (Vromman et al., 2021).

#### 2.5.2 Cell Culture

SW480 cells were cultured at 37°C, 0% CO_2_ in Leibovitz’s L-15 medium (31415-029, ThermoFisher). 10% foetal bovine serum (FBS) (F7524, Sigma) and 1% Penicillin-Streptomycin (10,000 U/mL) (15140122, ThermoFisher) were added to the medium.

#### 2.5.3 RNA Isolation

RNA was isolated from the cells using the miRNeasy Mini kit (217004, Qiagen) according to the manufacturer’s instructions, including an on-column DNase treatment (79254, Qiagen).

#### 2.5.4 RNase R Treatment and RT-qPCR

RNase R treatment and RT-qPCR were performed following a previously described protocol (Vromman et al., 2021).

## 3 Results

Here, we present CIRCprimerXL, a high-throughput and user-friendly circRNA primer design tool and web interface. CIRCprimerXL can be used to design circRNA primers for any species (using the command line version) and provides numerous user settings, including input template and amplicon size, SNP and secondary structure filtering, specificity evaluation, and multiple primers specifications.

### 3.1 Availability and Usage

CIRCprimerXL is implemented as a web application making it accessible through a web browser without the need for package or software installation.

**FIGURE 2:**
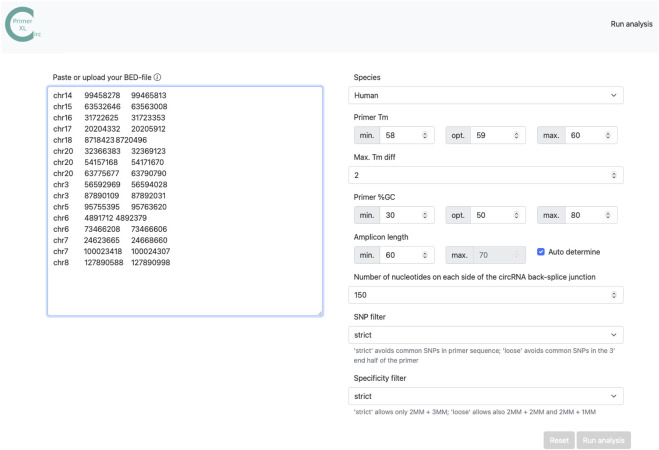
CIRCprimerXL web interface. CIRCprimerXL is implemented as a user-friendly web-interface, where multiple parameters (Table 2) can be adjusted.

The web interface of CIRCprimerXL (https://circprimerxl.cmgg.be) is an easy-to-use application to design primers for human, mouse, rat, zebrafish, *Xenopus tropicalis,* and *C. elegans* circRNAs (Figure 2). The parameters are described in detail in Table 2. The results are made available as multiple files, including a list containing the optimal primer pair for each circRNA, the complete list of all primer pairs that passed the filters, and a report showing primer design rates and reasons for failure.

**TABLE 2:** CIRCprimerXL web tool parameters. CIRCprimerXL takes multiple parameters to adjust the species, input template and amplicon size, GC ant Tm primer settings, SNP, and specificity evaluation.

Parameter	Default	Options
species	human	human, mouse, rat, zebrafish, *Xenopus tropicalis,* or *Caenorhabditis elegans*
		Of note, the SNP filter is only activated when designing primers for human circRNAs
splicing	yes	yes: the template sequence is spliced
		no: the template sequence is not spliced
template length	150 (which results in a total template length of 300 nucleotides)	any integer between 50 and 500
amplicon length	calculated based on template length	any integer between 50 and 500
primer GC settings	30-50-80 (minimum—optimum—maximum)	any integer between 15 and 85
primer Tm settings	58-59-60 (minimum—optimum—maximum)	any integer between 50 and 70
SNP filter	strict	strict: avoids common SNPs in primer design
		loose: avoids common SNPs in the 3′ end half of primer
off-targets filter	strict	strict or loose (see Table 1)

The Nextflow pipeline allows the user even more flexibility in primer design in addition to the options described for the web interface. First, the user can supply their own reference genomes and SNP database, enabling primer design for any species. Furthermore, the user can change the settings used by Primer3 for primer design (including the number of nucleotides overlapping between two primers, and the total number of primers tested per circRNA). Finally, the user can disable the upfront SNP and secondary structure flagging. The CIRCprimerXL pipeline is freely available on GitHub under an MIT license. Due to the license of some dependencies, CIRCprimerXL can only be used for non-commercial academic purposes. The license details can be found on the web interface and the GitHub page. CIRCprimerXL can easily be run locally or on a server or high-performance computer infrastructure. As CIRCprimerXL runs in a Docker container, the pipeline can be run on any operating system. The software, documentation, and test data can be found at: https://github.com/OncoRNALab/CIRCprimerXL.

### 3.2 Primer Design Success Rate

**FIGURE 3:**
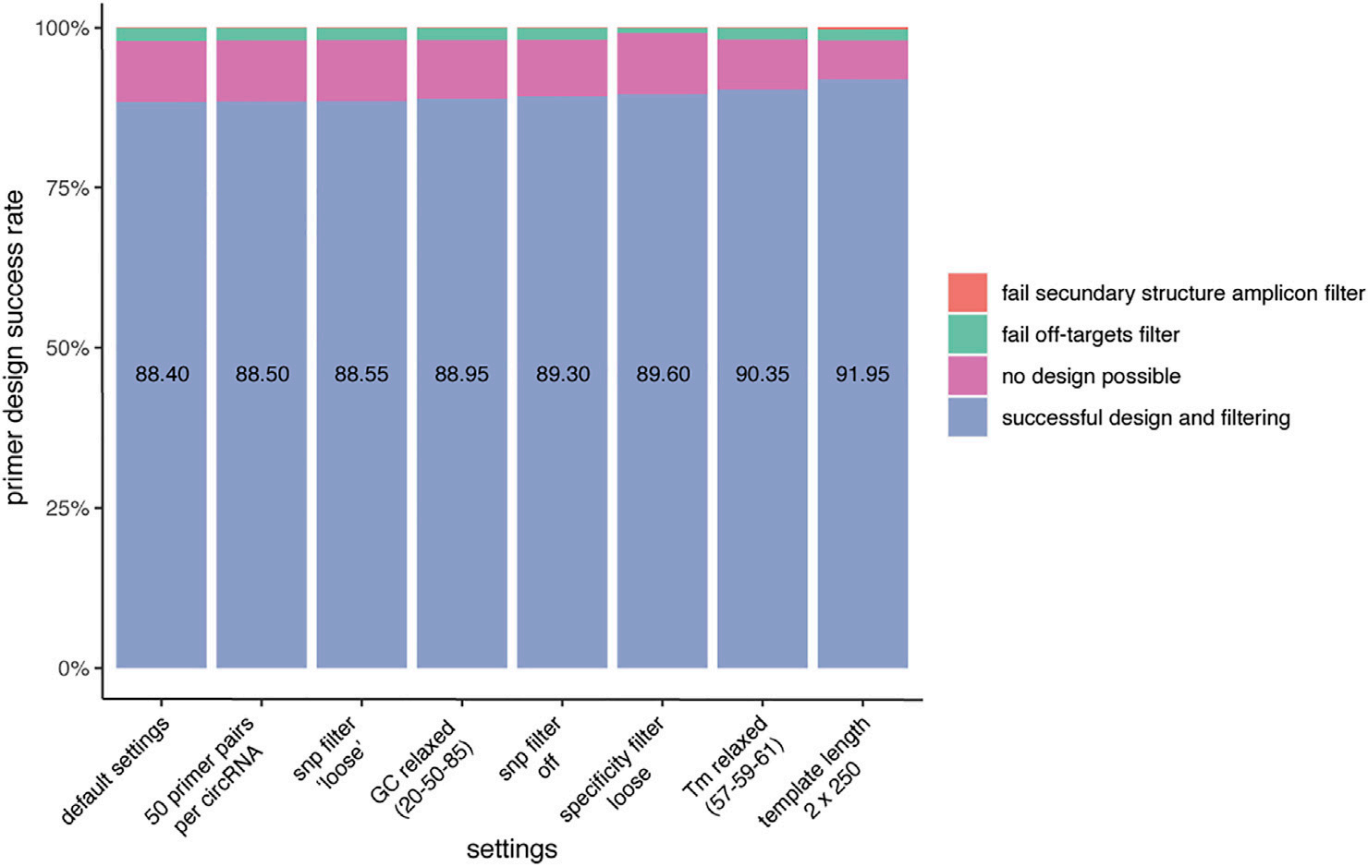
CIRCprimerXL has a high primer design success rate. When using default settings, CIRCprimerXL has a primer design success rate of almost 90% (here tested for 2000 circRNAs). This can be increased by optimizing the CIRCprimerXL parameters. The most improvement can be achieved by allowing a larger design space (template length of 500 instead of 300).

To investigate the primer design success rate, CIRCprimerXL was used to design primers for 2000 circRNAs detected by multiple different circRNA detection tools. Overall, a primer pair could be designed for 88.4% of the circRNAs using the default settings (Figure 3). The primer design success rate is dependent on multiple factors. For the majority of circRNAs for which no primer pair could be designed, Primer3 is not able to propose any suitable primer pair. This can be due to a too high/low GC content, a too high/low melt temperature, a long homopolymer (poly-x) sequence, high hairpin stability, and/or overlap with an excluded region (previously provided by the pipeline to avoid SNPs and secondary structures). The remaining circRNAs for which no primers could be designed are mostly due to the primers having predicted off-targets. Finally, a small number of primers are also rejected because of secondary structures in the primer binding sites of the amplicons. Adjusting different settings can increase the primer design success rate.

### 3.3 Run Time

Due to the use of Nextflow and Docker, CIRCprimerXL runs fast and is highly scalable (Table 3).

**TABLE 3:** CIRCprimerXL is efficient and can be used for large circRNA datasets. CIRCprimerXL duration times presented in this table are examples from single runs. The duration time can vary substantially based on the chosen parameters.

Number of input circRNAs	Run time examples	
	Standard laptop	HPC (40 GB memory, 16 CPUs)
2	2 min	33 s
20	7 min 43 s	1 min 12 s
200	1 h 2 min 58 s	9 min 37 s
2000	NA	2 h 47 min 03 s
15,000	NA	25 h 58 min 52 s

### 3.4 Assessment of PCR Amplification Efficiency of 20 circRNA Primer Pairs

**FIGURE 4:**
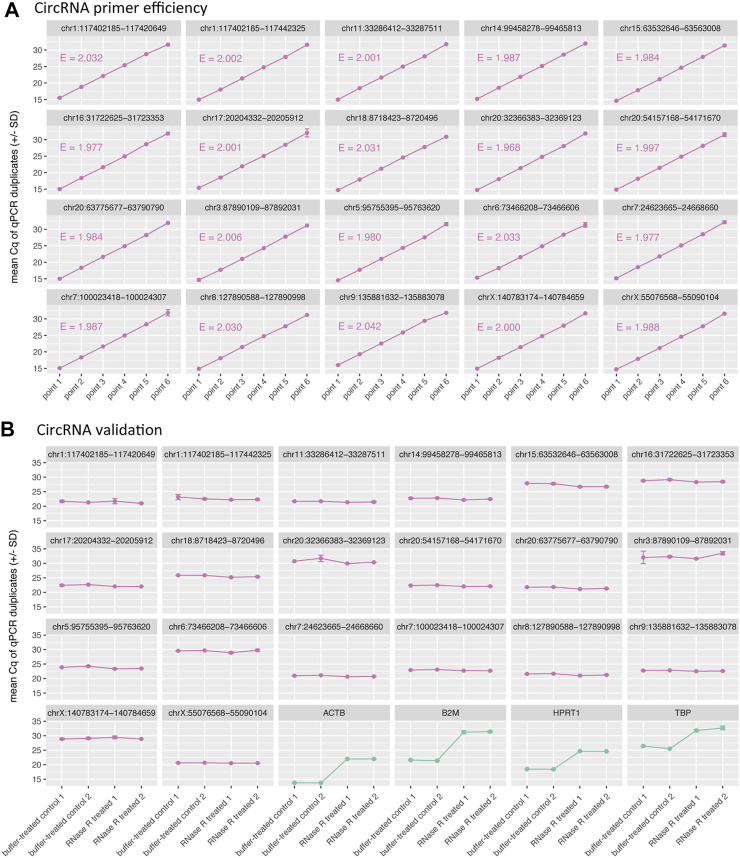
CIRCprimerXL primers are efficient and can validate circRNAs. **(A)** The efficiency (E) of the primers is assessed using a 6-point 10-fold dilution of a synthetic DNA template positive control. All primers showed an E-value between 1.9 and 2.1, which corresponds to efficiency between 90 and 110%. **(B)** These primers where then tested on SW480 total RNA (two treatment replicates, two qPCR replicates). The 20 circRNAs remained stable (similar Cq value) upon RNase R treatment, in contrast to the 4 linear control genes, that were degraded (increase in Cq value).

For empirical validation of CIRCprimerXL, 20 circRNAs were randomly selected and examined in detail. The PCR efficiency was tested on a dilution series of a synthetic DNA template positive control for each primer pair (Figure 4A). All primer pairs displayed an efficiency between 90 and 110%.

### 3.5 Measurement of 20 circRNAs in With RT-qPCR

To validate the circRNAs, RNA isolated from the SW480 cells was used. All 20 primers were measured by RT-qPCR on RNase R treated SW480 RNA, compared to a buffer-control SW480 RNA sample (Figure 4B). All circRNAs remained stable under RNase R treatment, in contrast to four linear control genes, that all (expectedly) show degradation (increase in Cq value).

## 4 Discussion

RT-qPCR combined with RNAse R treatment is currently the most straightforward method to validate circRNAs that have been detected in sequencing data. For this, circRNA-specific primer design is essential. Here, we present CIRCprimerXL, a high-throughput and user-friendly circRNA primer design tool and web interface. CIRCprimerXL can be used to design circRNA primers for any species (using the command line version) and allows the user numerous other settings, including input template and amplicon size, SNP, and secondary structure filtering, specificity evaluation, and multiple primers specifications. CIRCprimerXL has a primer design success rate of almost 90% with default parameters, which can be increased by adjusting the settings. CIRCprimerXL is efficient and can be used for large circRNA datasets. Finally, we validated the PCR efficiency of 20 circRNA primer pairs designed by CIRCprimerXL and showed how these primer pairs can quantify circRNAs in total RNA from SW480 cells.

## Data Availability

The original contributions presented in the study are included in the article/Supplementary Material, further inquiries can be directed to the corresponding author.
